# Supplementary figures and images for: Integrated metabolome and transcriptome revealed the flavonoid biosynthetic pathway in developing Vernonia amygdalina leaves
Source: PeerJ. 2021 Apr 26;9:e11239. doi: 10.7717/peerj.11239 (PMC8083182; doi:10.7717/peerj.11239)

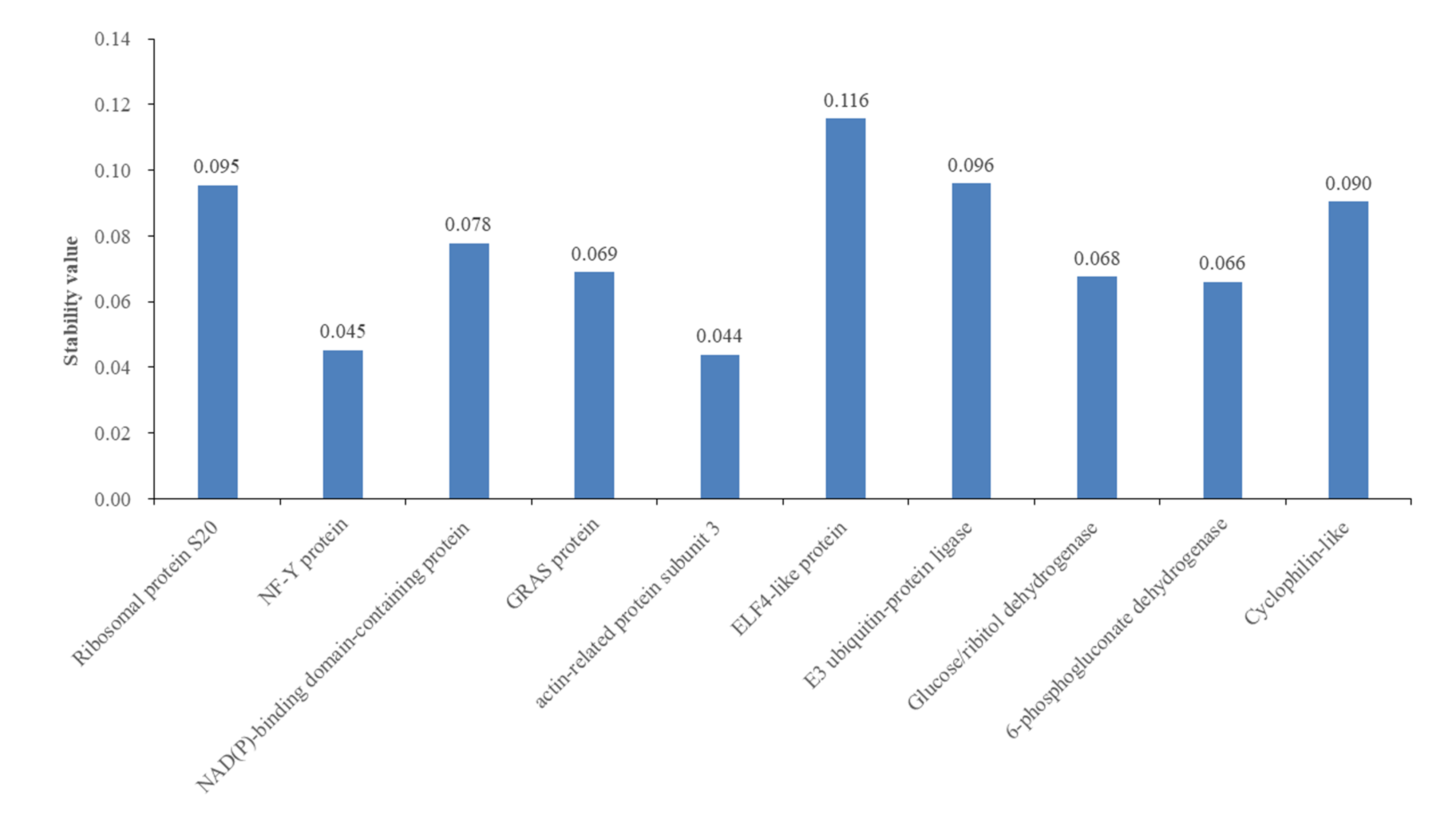

Supplement: Supplemental Information 1 [file peerj-09-11239-s001.png]

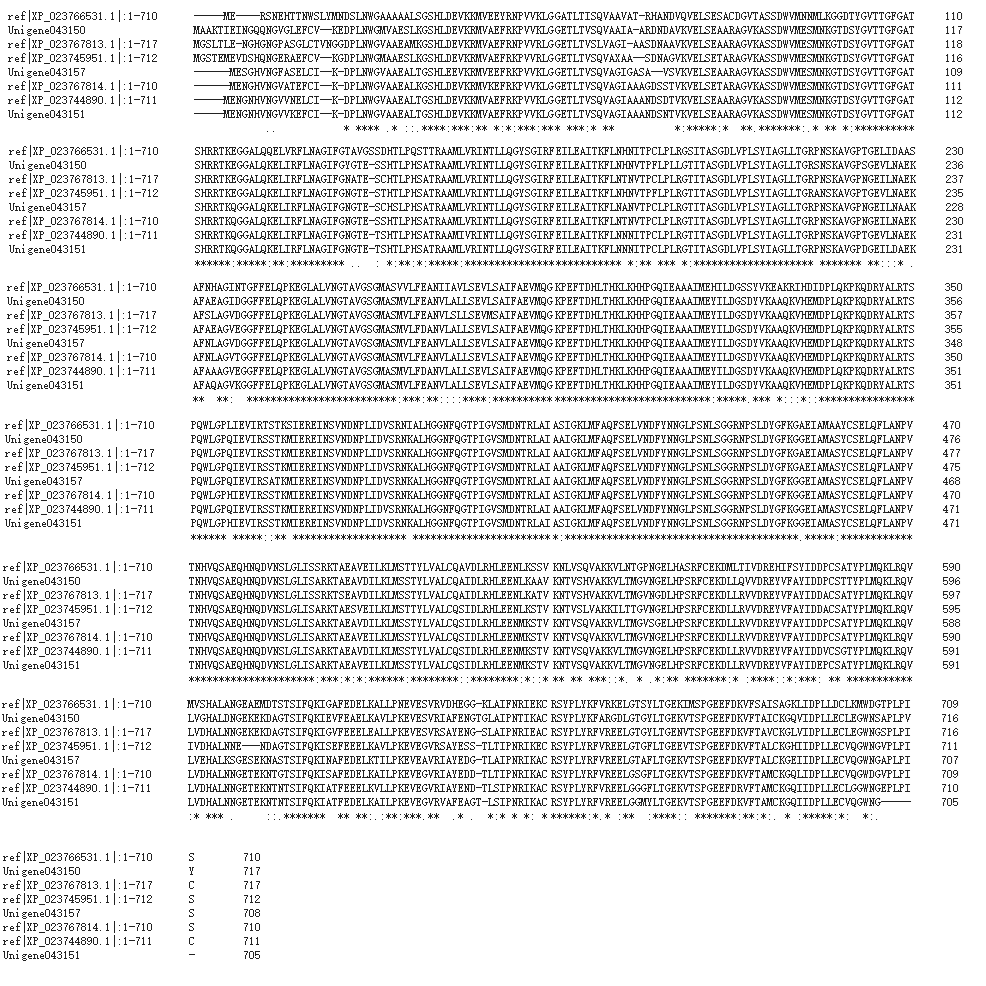

Supplement: Supplemental Information 2 [file peerj-09-11239-s002.png]

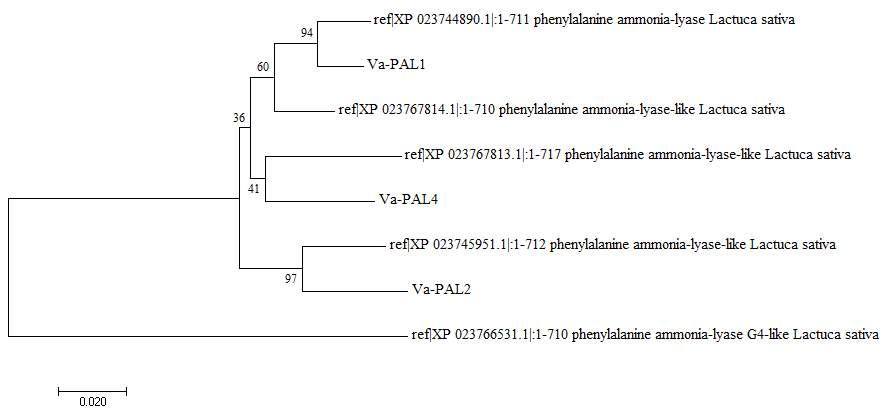

Supplement: Supplemental Information 3 [file peerj-09-11239-s003.png]
