# Supplementary material for: Integrated metabolome and transcriptome revealed the flavonoid biosynthetic pathway in developing Vernonia amygdalina leaves
Source: PeerJ. 2021 Apr 26;9:e11239. doi: 10.7717/peerj.11239 (PMC8083182; doi:10.7717/peerj.11239)
Supplement: Supplemental Information 7 [file peerj-09-11239-s007.docx]

Table S4 The results of transcriptome assembly completeness by BUSCO v4.1.2

|  | Complete BUSCOs (C) | Complete and single-copy BUSCOs (S) | Complete and duplicated BUSCOs (D) | Fragmented BUSCOs (F) |
| --- | --- | --- | --- | --- |
|  | 1808(77.7%) | 1745(75.0%) | 63(2.7%) | 185(8.0%) |
